# Upper-Arm Photoplethysmographic Sensor with One-Time Calibration for Long-Term Blood Pressure Monitoring

**DOI:** 10.3390/bios13030321

**Published:** 2023-02-25

**Authors:** Ching-Fu Wang, Ting-Yun Wang, Pei-Hsin Kuo, Han-Lin Wang, Shih-Zhang Li, Chia-Ming Lin, Shih-Chieh Chan, Tzu-Yu Liu, Yu-Chun Lo, Sheng-Huang Lin, You-Yin Chen

**Affiliations:** 1 Department of Biomedical Engineering, National Yang Ming Chiao Tung University, No. 155, Sec. 2, Linong St., Taipei 112304, Taiwan; 2 Biomedical Engineering Research and Development Center, National Yang Ming Chiao Tung University, Taipei 112304, Taiwan; 3 Material and Chemical Research Laboratories, Industrial Technology Research Institute, No. 195, Sec. 4, Chunghsing Rd., Hsinchu 310401, Taiwan; 4 Department of Neurology, Hualien Tzu Chi Hospital, Buddhist Tzu chi Medical Foundation, No. 707, Sec. 3, Zhongyang Rd., Hualien 970473, Taiwan; 5 Department of Neurology, School of Medicine, Tzu Chi University, Hualien 97004, Taiwan; 6 Microlife Corporation, 9F, No. 431, Ruiguang Rd., Taipei 114063, Taiwan; 7 The Ph.D. Program in Medical Neuroscience, College of Medical Science and Technology, Taipei Medical University, No. 250, Wu-Xing St., Taipei 11031, Taiwan; 8 Medical Device Innovation and Translation Center, National Yang Ming Chiao Tung University, Taipei 112304, Taiwan

**Keywords:** cuffless blood pressure monitor (cuffless BPM), upper-arm photoplethysmography (upper-arm PPG), wearable device, medical regulation, long-term monitoring

## Abstract

Wearable cuffless photoplethysmographic blood pressure monitors have garnered widespread attention in recent years; however, the long-term performance values of these devices are questionable. Most cuffless blood pressure monitors require initial baseline calibration and regular recalibrations with a cuffed blood pressure monitor to ensure accurate blood pressure estimation, and their estimation accuracy may vary over time if left uncalibrated. Therefore, this study assessed the accuracy and long-term performance of an upper-arm, cuffless photoplethysmographic blood pressure monitor according to the ISO 81060-2 standard. This device was based on a nonlinear machine-learning model architecture with a fine-tuning optimized method. The blood pressure measurement protocol followed a validation procedure according to the standard, with an additional four weekly blood pressure measurements over a 1-month period, to assess the long-term performance values of the upper-arm, cuffless photoplethysmographic blood pressure monitor. The results showed that the photoplethysmographic signals obtained from the upper arm had better qualities when compared with those measured from the wrist. When compared with the cuffed blood pressure monitor, the means ± standard deviations of the difference in BP at week 1 (baseline) were −1.36 ± 7.24 and −2.11 ± 5.71 mmHg for systolic and diastolic blood pressure, respectively, which met the first criterion of ≤5 ± ≤8.0 mmHg and met the second criterion of a systolic blood pressure ≤ 6.89 mmHg and a diastolic blood pressure ≤ 6.84 mmHg. The differences in the uncalibrated blood pressure values between the test and reference blood pressure monitors measured from week 2 to week 5 remained stable and met both criteria 1 and 2 of the ISO 81060-2 standard. The upper-arm, cuffless photoplethysmographic blood pressure monitor in this study generated high-quality photoplethysmographic signals with satisfactory accuracy at both initial calibration and 1-month follow-ups. This device could be a convenient and practical tool to continuously measure blood pressure over long periods of time.

## 1. Introduction

Hypertension is a major risk factor for various cardiovascular diseases [1,2,3]. It is estimated that more than one billion adults worldwide have hypertension, of whom 46% are unaware of their condition and only 21% effectively manage it [2,4]. The accurate diagnosis and effective management of hypertension rely on accurate blood pressure (BP) measurements; the former is typically based on repeated in-office BP measurements or out-of-office BP measurements through ambulatory and/or home BP monitoring [2,5]. Home blood pressure monitors (BPMs) are more widely used than ambulatory BPMs thanks to their convenience, lower cost, easier accessibility, and ability to monitor long-term BP [2]. Self-monitoring at home also enables the diagnosis of white-coat and masked hypertension [2] while improving patient adherence to treatment plans for hypertension [2,6,7,8].

Advances in mobile technology have resulted in the widespread use of health-related wearables and smartphone apps. Wearable cuffless BPMs, such as the Aktiia bracelet, Samsung Galaxy Watch, and Biobeat chest/wrist monitor, as well as smartphone-based BP-measuring apps such as the Riva digital app, AlwaysBP, and Opti BP, have gained popularity in recent years [9,10,11,12,13,14,15,16]. These cuffless BPMs use optical sensors to track changes in BP. Cuffless BP measurement devices measure wrist pulse waveforms by combining photoplethysmography (PPG) [17] with an electrocardiogram (ECG) and the oscillometric finger-pressing method [18] to determine the pulse wave transit time (PWTT) [19] between the peaks of the PPG and ECG signals. However, newer technologies rely on only PPG signals to estimate BP. In addition to simplifying the complexity of the device, this eliminates the need for an additional ECG sensor, enabling continuous BP monitoring for 24 h or longer without disrupting daily life and sleep [20,21,22,23,24].

The cuffless PPG-type BPM estimates BP by measuring the finger, wrist, and upper arm. Finger PPG signals are more reliable estimators of changes in BP and are therefore better suited for continuous monitoring because they are less affected by movement and positioning than wrist or arm PPG signals. In addition, finger PPG signals may be more accurate in certain populations, such as elderly people or those with peripheral artery disease because the finger arteries may be more responsive to changes in BP [25]. However, such devices worn on the fingers may inconvenience users in daily life and can generate motion artifacts in the fingers that confound BP measurements from PPG signals [26]. Still, wrist-type BPMs are also widely used [24]. However, according to Hartmann et al., the PPG signal measured at the wrist-upper, the position at which wrist-type PPG measuring devices are typically worn, is less capable of representing the PPG pulse waveform than the signal measured by arm-type devices [25]. In addition, upper-arm PPG BP-measuring devices are less likely to be affected by motion artifacts [27]. Reducing movement interference also enhances the quality of PPG signals and therefore BP estimations [28]. The wrist is relatively far from the heart, and wrist-type BPMs can generate errors (e.g., as high as 10 mmHg) if the height of the wrist is not level with the heart during BP measurement [29].

The exponential Gaussian process regression (expGPR) has proven to be the most suitable machine-learning (ML) model for PPG physiological data [30]. The upper-arm, cuffless PPG BPM developed in this research adopts the expGPR model architecture with the fine-tuning method [31,32]. Previous research has combined the expGPR machine-learning model with a kernel (in this case, the exponential kernel function) to calculate BP; the Gaussian process (GP) in this model could be used to represent the distribution of a function. Currently, a common ML approach is to parameterize a function and then use the generated parameters to avoid distributed representation. However, the expGPR model used in this study differed in that it directly modeled the function to generate a nonparametric model. One of the main advantages of this method was that it could not only simulate any black-box function but also model uncertainty. This quantification of uncertainty with the help of a GP enabled us to explore the data regions that were least likely to be efficiently trained when more data were allowed to be requested, thereby improving the accuracy of BP estimation. Despite the good performance of this model, its memory capacity and computational speed must increase with the size of the training data and the kernel design, a key issue for embedded systems.

A BPM must be regularly and clinically validated according to a standard protocol before it can be used for diagnostics [33]. Although several commercially available cuffless BPMs have achieved satisfactory clinical validation results according to the ISO standard [15,16,34,35], most of these cuffless BPMs still require an initial calibration by using a traditional cuffed BPM and therefore depend on their accuracy. In addition, the systolic BP (SBP) and diastolic BP (DBP) measured by cuffless BPMs are estimated by using mathematical modeling based on the initial calibration with the cuffed BPMs, and they often require regular recalibration. For instance, a recalibration every four weeks is recommended for the Samsung Galaxy Watch and Aktiia bracelet [9,10]. Given these limitations, a validation protocol for cuffless BPMs should assess not only the initially calibrated BP measurements but also the changes in uncalibrated BP values at different time points after calibration [10].

The purpose of this study was to validate the accuracy of a newly developed upper-arm, cuffless PPG BPM according to the Association for the Advancement of Medical Instrumentation (AAMI)/European Society of Hypertension (ESH)/International Organization for Standardization (ISO) 81060-2 universal standard [33]. In addition, the long-term performance of the upper-arm, cuffless BPM was evaluated weekly for 1 month on the basis of recordings from the uncalibrated BP measurements. The upper-arm measurement design, located closer to the heart, was less susceptible to interference with the PPG signal that is due to significant body movement. We improved model performance with a smaller data set and model size by using the fine-tuning method to retrain the model. The BP measured by this device is expected to not only satisfy the accuracy requirements outlined in the ISO 81060-2 standard but also maintain accuracy without the need for continuous calibration.

## 2. Materials and Methods

### 2.1. Participants from Clinical Trial

The institutional review board (IRB) of Hualien Tzu Chi Hospital, Buddhist Tzu Chi Medical Foundation (IRB No. IRB111-206-B), approved the study. Informed consent was obtained from all participants, to whom the study protocol was fully explained. In total, 34 participants (aged 20 years and above) were recruited, and 30 of the 34 eligible participants were divided into predefined BP ranges on the basis of their reference BP (Table 1). The reference BP range was divided into three groups: hypertension (SBP/DBP ≥ 130/80 mmHg), normotension (90/60 mmHg ≤ SBP/DBP < 130/80 mmHg), and hypotension (SBP/DBP < 90/60 mmHg). Notably, the current study was designed to assess the long-term performance of an upper-arm, cuffless BPM and did not meet the international standard AAMI/ESH/ISO 81060-2 protocol with respect to the required number of participants and BP distribution stratification [33]. Participants who were pregnant or who had arrhythmia or a history of heart failure or heart attack were excluded. Four participants were excluded; one was excluded because of AFib, and the others because of interruptions in follow-up.

### 2.2. Upper-Arm Photoplethysmographic Sensor as a Cuffless Blood Pressure Monitor

**Figure 1:**
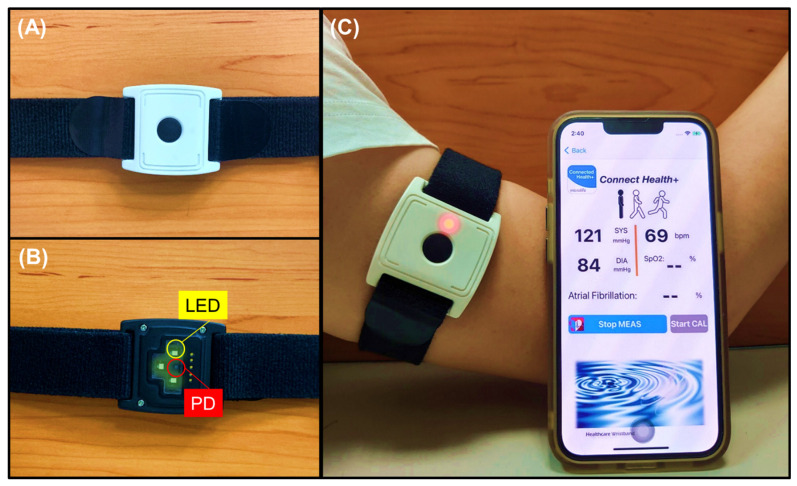
Our proposed cuffless BPM, based on the upper-arm reflective PPG detection. (**A**) The photography for the top view of the cuffless BPM, which presented a simple user interface with a power switch button and a LED-operated indicator. (**B**) The bottom view of the device showed the PPG hardware, consisting of three green LED elements and a PD arranged in a T-shape configuration, constructed with optical baffles to shade interferences from ambient light sources. (**C**) The BPM was worn on the upper arm, providing a cuffless BP measurement that was based on the reflective PPG signals. The measured data were wirelessly transmitted to a smartphone via the Bluetooth communication protocol and displayed the corresponding heart rate, SBP, DBP, and current posture on the app.

We designed a wearable device as a cuffless BPM, called the WatchBP O3 wearable (Microlife Corporation, Taipei, Taiwan), which could be worn on the upper arm and measure BP; its performance was evaluated during a 5-week BP calibration period. The WatchBP O3 wearable used a low-power embedded architecture with an ARM Cortex-M4 microcontroller (nRF52832, Nordic Semiconductor, Oslo, Norway) as its computing core. The processor had a maximum operating frequency of 64 MHz with 512 KB cache memory and 64 KB random access memory, and it supported the Bluetooth low-energy (BLE) communication protocol. The top of the device included a power switch button and a light-emitting diode (LED) operation indicator light (Figure 1A).

On the other side closest to the skin (the bottom of the device), there were three green light-emitting diode (LED) elements (CT DBLP31.12, OSRAM Opto Semiconductors GmbH, Regensburg, Germany) and a photodiode (PD) phototransistor (SFH 2704, OSRAM Opto Semiconductors GmbH, Germany). The optical sensing module under this configuration was arranged in a T shape (Figure 1B). The PD was in the middle of the three LEDs and was used to receive the PPG signal reflected by the LED. The WatchBP O3 wearable also had a 6-axis inertial measurement unit sensor (BMI270, Bosch Sensortec, Gerlingen, Germany) to filter out noise from the signal to stabilize the PPG signal and clarify the characteristic information. It was also used to recognize posture during walking, standing, sitting, sleeping, and resting. The built-in battery had a capacity of 160 mAh and could operate for a long time. The device could be fixed to the upper arm by using a 20 mm wide armband (two lengths, 34 cm and 42 cm, were available for different arm circumferences). The BP measurement function of the WatchBP O3 wearable could be paired with a BP application (BP App), developed by Microlife Corporation through BLE. Measurement would begin after pairing. Finally, the results were transmitted to the smartphone for storage and could be displayed in the BP App, which displayed heart rate, SBP, DBP, and current posture (Figure 1C).

**Figure 2:**
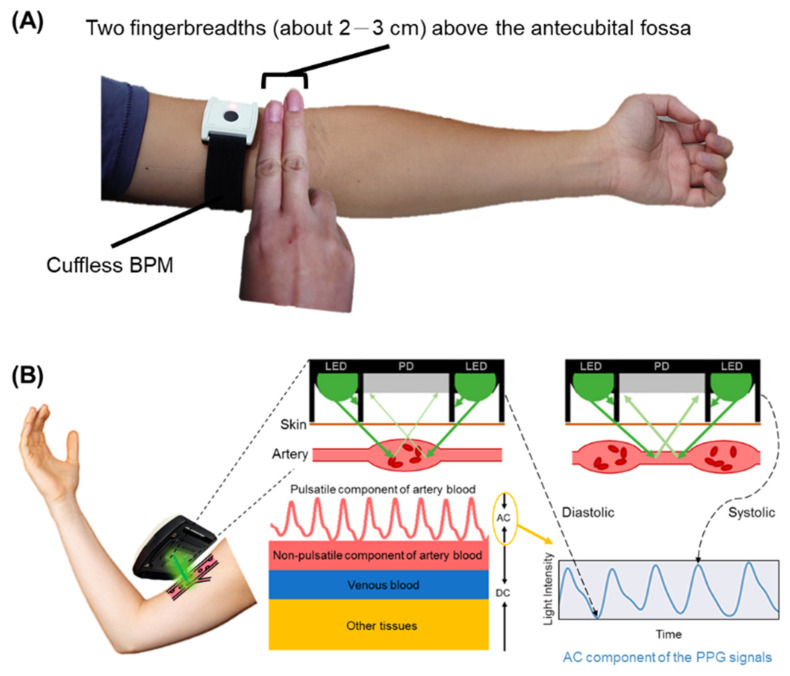
The optimal location for the cuffless BPM and theoretical basis of BP estimation from the reflective PPG signals. (**A**) The cuffless BPM could be located two fingerbreadths (about 2–3 cm) above the antecubital fossa by feeling the bicep tendon in the area of the antecubital fossa. (**B**) The schematic of the cuffless BPM device attached onto the upper-arm skin to acquire reflective PPG signals. The acquired PPG signals comprised an AC (pulsatile) and a DC (slowly varying) component. The AC component was attributed to changes in the blood volume synchronous with each heartbeat, whereas the DC component was related to respiration, tissues, and average blood volume. The corresponding working principle of the reflective PPG sensor involved capturing the AC component of the PPG signals, which defined approximate features, such as DBP, represented by valleys, and SBP, represented by peaks.

In this study, the reflected PPG signal was acquired from PD, receiving the green LED light reflected from the blood and tissue and detecting the difference in the intensity of the reflected light absorbed by the blood and tissue, which could be used to estimate BP on the basis of a machine-learning algorithm. The periodic fluctuation of PPG amplitude reflected the changes in blood volume of cutaneous microvascular bed during heart-beating cycle. The PPG signal consisted of (1) a DC signal that detected optical signal reflected by tissues, bones, and muscles, as well as the average blood volume in arteries and veins, and (2) an AC signal indicating the change in blood volume between the systolic and diastolic periods of the heart-beating cycle. A PPG signal that reflects the characteristics of blood flow was obtained by extracting the AC signal. The cuffless BPM is positioned on the inner arm, near the brachial pulse, approximately 2 cm medial from the biceps tendon in the antecubital fossa and 2–3 cm above it (Figure 2A) [36] The LED illuminated the skin and tissue close to the upper arm during BP measurement, and the PD embedded in the center of the back plate received the PPG signal from the skin tissue blood volume changes (Figure 2B).

### 2.3. Machine-Learning Framework for an Embedded Optimal Design on the Fine-Tuning Method

Our team previously pretrained the expGPR model using a BP database of PPG signals measured at the wrist [30]. However, we measured the PPG signal sampled at a 256 Hz sampling rate at the upper arm in this study. We used the fine-tuning method to directly optimize the expGPR model and avoid retraining it with the added PPG signal from the upper arm, which would create a model too large for the microcontroller (MCU) to handle because of the increased computation and memory capacity.

There were very little or no public data available on upper-arm PPG signals, and the BP waveform characteristics of upper-arm PPG signals were highly correlated with those of wrist PPG signals. Therefore, we directly extracted 10 PPG waveform characteristics per 15-s interval from the previous study, which included the following: waveform parameters and time-related parameters such as the systolic area over total area, diastolic area over total area, systolic area over pulse amplitude, diastolic area over pulse amplitude, maximal amplitude over time as maximal slope, systolic time, diastolic time, and mean peak-to-peak interval. The pretrained model parameters were used as the initial parameter values for the pretrained model. We then re-trained the model using the new upper-arm PPG database collected in this study, where we adjusted the kernel parameters to optimize the pretrained model. The kernel adjustment parameters were the kernel scale and the signal standard deviation. We first fixed the initial values of these two parameters to the values calculated when only wrist PPG signals were added to the training data. The calculation method is as follows:

The kernel scale is defined as that through which the software searched among real values in the range $\left[0.001,\;1\right]\times X_{MaxRange}$. $X_{MaxRange}$ is expressed in Equation (1):

(1) $$X_{MaxRange}=\max\left(\max\left(X\right)-\min\left(X\right)\right)$$

where X is the predictor data by feature matrix. The signal standard deviation S is defined in Equation (2) as N represents the number of predictor data, and *μ* represents the mean value of predictor data, as in Equation (3):

(2) $$S=\sqrt{\frac{1}{N-1}\overset{N}{\underset{i=1}{\sum }}{\left|X_{i}-\mu \right|}^{2}}$$

(3) $$\mu =\frac{1}{N}\overset{N}{\underset{i=1}{\sum }}X_{i}$$

Through this formula, we fixed the kernel scale and the signal standard deviation of the pretrained model to 11.9 and 9.6, respectively. This reduced the size of the training data feature matrix from 54.2 KB (444,480 bits, calculated as 1389 data length × 10 features × 32 bits using float) to 43.4 KB (355,520 bits, calculated as 1111 data length × 10 features × 32 bits using float) and the training time from 16.9 seconds to 1.6 seconds. Therefore, optimizing and validating the kernel function not only improved the overall accuracy of the model but also required less time and memory. This also prevented the model from overfitting as a result of excessive data.

### 2.4. Integration of an Autocalibrated System for a Cuff-Based and Cuffless Blood Pressure Monitor through a Smartphone

**Figure 3:**
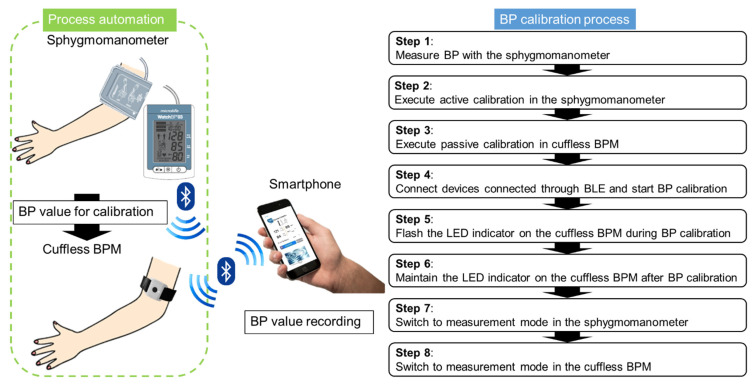
Autocalibration process for the cuffless BP measurement via a cuff-based (sphygmomanometer) monitor. To take the cuffless BP measurement the first time, a user starts to operate a BP cuff with a sphygmomanometer to manually measure BP. Following manual BP measurement, the succeeding procedures of BP calibration are automatically executed by the cuffless BPM.

The WatchBP O3 wearable could also be paired with the upper arm BPM as a sphygmomanometer, called Microlife WatchBP O3 (Microlife Corporation, Taipei, Taiwan), to calibrate BP measurements and improve their accuracy. The calibration mode required the WatchBP O3 wearable, WatchBP O3, and smartphone to be placed within the range of BLE transmission. The calibration mode of the WatchBP O3 and WatchBP O3 wearable was activated through the BP App, and BP was measured using the WatchBP O3. After the measurement was complete, the data were automatically transmitted to the WatchBP O3 wearable to calibrate the model. Such autocalibration could reduce the likelihood of manually inputting incorrect data and hence increase the precision of subsequent BP estimations. Figure 3 depicts this process, which was divided into eight steps.

The BP App was a cuffless BPM based on mobile health (mHealth) applications and a specific wearable device to prevent and manage chronic diseases. The BP App display provided the following information: SBP and DBP, pulse rate, posture, date, and battery status. The BP App had two modes: default and calibration. The former reported a single BP measurement, whereas the latter indicated a BP value used for device calibration (Figure 3). It was recommended to perform the BP measurement again if the signal quality was poor, which might have been due to arm movements or loose contact. A change in PPG characteristics indicated a potential change in BP or personal variations. Artificial intelligence and ML algorithms were used to calculate the change in SBP and DBP on the basis of the nonlinear association between BP and PPG characteristics. An initial BP calibration using a cuffed BPM was required because PPG characteristics can estimate only changes in BP. A must-have feature of the device was its ability to accurately measure BP in calibration mode.

### 2.5. Validation of ISO Protocol Based on One-Time Calibration for Long-Term Blood Pressure Monitoring

The study consisted of two parts: the main analysis validated the accuracy and assessed long-term performances of the upper-arm, cuffless PPG BPM, whereas the secondary analysis evaluated the qualities of PPG signals obtained from the upper-arm and wrist-type PPG BPMs.

**Figure 4:**
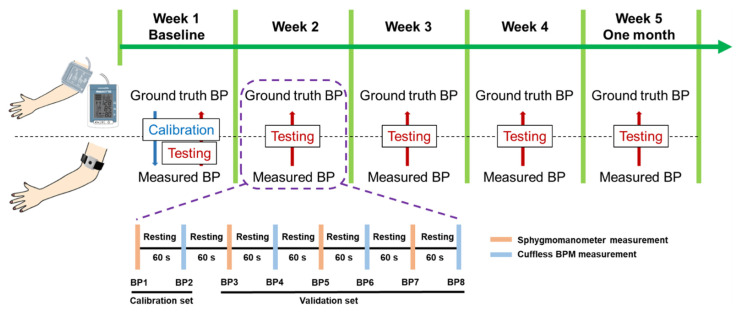
The measurement sequences for validating the upper-arm, cuffless PPG BPM devices by following the AAMI/ESH/ISO 81060-2 protocol for the validation of cuff-based BP measurement devices. These sequences began with baseline measurements using the reference method and the test device, followed by the validation set. An initial calibration to the reference method was a prerequisite to validating the upper-arm, cuffless PPG BPM. After the first calibration, additional validations were performed at different time points over 1 month (week by week) for a single calibration.

First, the study protocol followed the ISO 81060-2 standard, with an additional four BP measurements, over a 1-month period to assess the long-term performance values of the cuffless BPM (i.e., the Microlife WatchBP O3 wearable should be calibrated at least every 4 weeks) (Figure 4). Three well-trained observers who familiarized themselves with the test device and protocol measured BP. To ensure the accuracy of PPG signals, it was crucial to maintain adequate contact force between the skin and cuffless BPM during testing. This was because the contact force affected both the relative motion between the sensor and the measuring site, as well as the arterial geometry [37]. To ensure consistent contact pressure between the cuffless BPM and the skin, all observers were instructed to secure the WatchBP O3 strap snugly enough to stay in place but not too tightly to cause discomfort. This specific level of contact pressure has previously been demonstrated to produce accurate heart-rate signals when using the PPG sensor [38].

The reference measurement for this study was recorded using the WatchBP O3 sphygmomanometer with a digital screen and the cuffs provided by the device. The reference sphygmomanometer was calibrated by using the same WatchBP O3 prior to the study. BP was measured in this study by using the same-arm sequential method, according to the AAMI/ESH/ISO 81060-2 protocol [33]. The measurements were alternately taken using the reference sphygmomanometer (i.e., WatchBP O3) and the test device (i.e., WatchBP O3 wearable). Two observers who were blinded to each other’s readings performed the measurements by using the standard method and the test device. A third observer served as a supervisor who also measured BP by using the test device. All measurements were performed on the nondominant arm of the subject while they comfortably sat in a quiet, temperature-controlled room (20–22 °C [68–72 °F]). The subject was asked to remain silent throughout the procedure. After resting for 10 minutes, BP was measured with the forearm supported and the cuff positioned at heart level, as recommended in the guidelines [6]. A baseline measurement was recorded by using the reference sphygmomanometer before validation. The BP measurements were then alternated between the reference sphygmomanometer and the cuffless BPM, resulting in eight readings for each subject: four readings with the reference standard sphygmomanometer (labeled as BP1 (‘calibrated’), BP3, BP5, and BP7) and four readings with the BP App (labeled as BP2 (‘calibration’), BP4, BP6, and BP8). There was a minimum interval of 60 seconds between each measurement. The same procedure without calibration (i.e., BP1 and BP2 were not required) was repeated in follow-up sessions from week 2 to week 5 (Figure 4).

To compare the quality of the PPG signals obtained from the upper-arm and wrist-type BPMs, the BP values of all 30 participants at week 1 were measured by using the upper-arm, cuffless PPG BPM and the wrist-type PPG BPM [30]. The chronology was randomized.

### 2.6. Statistical Analysis of Blood Pressure Estimations

To assess PPG signal quality, 120 PPG pulses measured by both upper-arm and wrist-type BPMs from each subject were randomly selected and assigned to one of the three quality levels by three raters, defined as follows [39]:

1. Fair—systolic and diastolic peaks cannot be easily distinguished from noise.
2. Good—the systolic peak is clearly detectable, but the diastolic peak is not.
3. Excellent—systolic and diastolic peaks are both clearly detectable.

A paired *t*-test was used to compare the differences in the PPG signals obtained from the upper-arm and wrist-type device in each of the three quality levels. All analyses were performed using SPSS 20.0 (SPSS Inc, Chicago, IL, USA).

For primary analysis, each of the three BP readings (except for the calibration reading) taken from the upper-arm, cuffless PPG BPM was compared with those taken from the reference BP (i.e., average of BP3 was compared with BP4, average of BP5 was compared with BP6, and average of BP7 was compared with BP8), generating 90 pairs of measurements per week.

Two international guidelines, the ISO 81060-2 standard and the British Hypertension Society (BHS) guideline, were used to evaluate the accuracy and long-term performance values of the upper-arm, cuffless PPG BPM. The mean and standard deviation (SD) of the difference between the readings from the test and reference device were calculated to determine whether the results met the passing criteria of the ISO 81060-2 standard:

(1) Criterion 1—the mean ± SD of the SBP and DBP ≤5 ± ≤8.0 mmHg.

(2) Criterion 2—the SD ≤6.89 mmHg and ≤6.84 mmHg, respectively, for SBP and DBP [33].

The standard accuracy criteria of the BHS were defined as follows for the cumulative percentage in 5, 10, and 15 mmHg, with four grades: Grade A (≤5, 60%; ≤10, 85%; and ≤15, 95%), Grade B (≤5, 50%; ≤10, 75%; and ≤15, 90%), Grade C (≤5, 40%; ≤10, 65%; and ≤15, 85%), and Grade D (worse than Grade C). A Pearson correlation analysis and a Bland–Altman analysis were used to assess agreement between the test and reference devices. In addition, the BP values of subjects with Fitzpatrick skin type II and type III were compared by using the Wilcoxon rank sum test to further examine the impact of skin phototype on BP measurements.

## 3. Results

### 3.1. Participant Demographic

**Figure 5:**
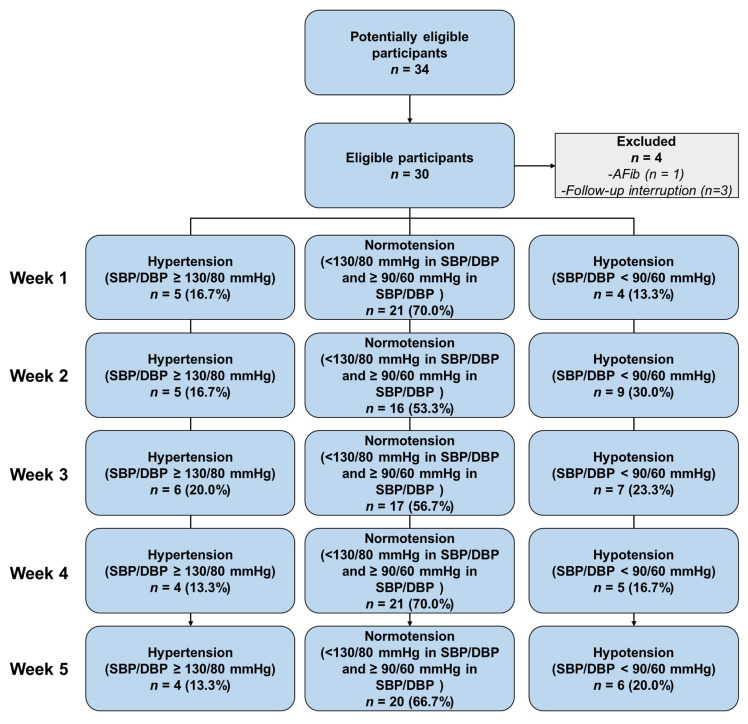
Flowchat of 1-month follow-up, based on the STARD 2015 guidelines. Eligible participants without AFib and follow-up interruption were divided into three groups: hypertension, normotension, and hypotension. Weeks 1–5 showed similar partitions (hypertension: 13.3–20%; normotension: 53.3–70%; hypotension: 13.3–23.3%), validating the long-term data set.

In total, 34 subjects were recruited for this study. Four subjects were excluded from the analysis, of whom one had a history of atrial fibrillation, and the other three failed to complete the follow-up sessions. This resulted in 30 subjects (age: 26.3 ± 5.3 years; sex: female 11 [36.67%], male 19 [63.33%]) and 90 pairs of valid BP readings per week. The average upper-arm circumference was 27.2 ± 2.8 cm, with a range of 22.0–31.0 cm, covering the full range of the standard M–L cuffs used. The participants’ demographics are shown in Table 1. Figure 5 shows the number of subjects in each of the BP categories (i.e., hypotension, normotension, and hypotension) according to their entry BP for weekly evaluation. The reference BP readings for SBP and DBP met the distribution of the predefined BP ranges as specified in the ISO 81060-2 standard.

### 3.2. Comparison of PPG Signals from Upper-Arm, Cuffless PPG BPM and Wrist-Type PPG BPM

**Figure 6:**
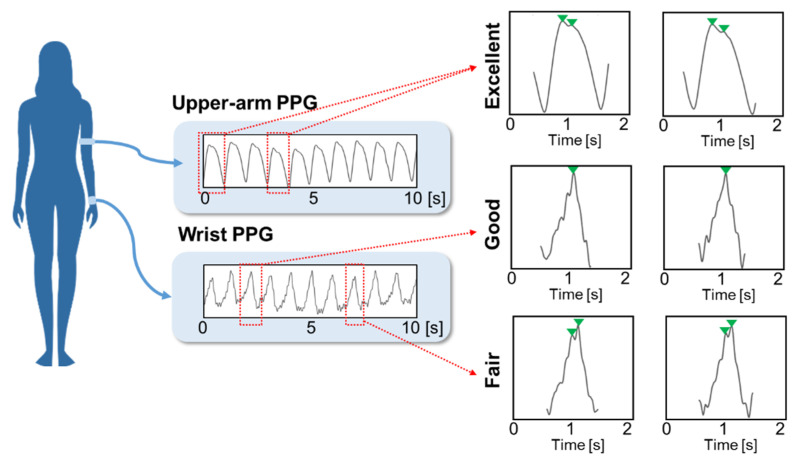
Representative PPG signals measured at the upper arm and wrist, respectively, and compared with classifications as fair, good, and excellent in terms of signal quality. These PPG signals can be simultaneously obtained from various body locations. At the excellent level, the systolic peak and diastolic peak (green triangle upside down) can be clearly detected, and the amplitude of the diastolic peak is lower than the amplitude of the systolic peak. At the good level, only one peak, similar to the systolic peak, can be detected. At the fair level, the PPG signal contains some noise that may affect peak detection.

The characteristics of the PPG signals and their quality levels measured by the upper-arm and wrist-type BPMs from the same subject are shown in Figure 6. An excellent PPG signal was characterized by distinguishable systolic and diastolic peaks.

**Figure 7:**
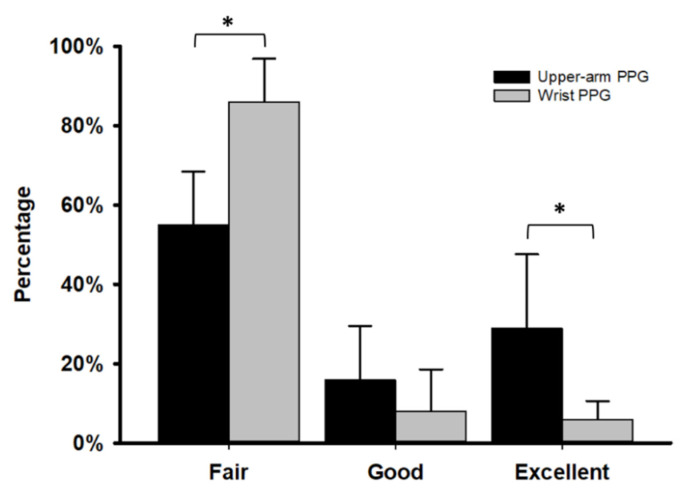
Distribution of the three quality levels in PPG signals measured at the upper arm (black column) and wrist (gray column). The bar plot represents mean and SD (error bars). The upper-arm PPG generally had a higher proportion of excellent (29 ± 16%) and good (16 ± 12%) pulses, while the wrist PPG had a higher proportion of fair (86 ± 9%) pulses. The difference in proportions between the two quality levels (i.e., fair (*p* = 0.029) and excellent (*p* = 0.029)) was statistically significant (* *p* < 0.05).

Figure 7 shows the quality level differences in the PPG signals obtained from the upper-arm, cuffless PPG BPM and the wrist-type PPG BPM. In general, up to 45% of the PPG signals measured by the upper-arm, cuffless PPG BPM could be categorized as good (16% ± 4%) to excellent (29% ± 6%), and the remaining signals were fair (55% ± 4%). On the other hand, less than 15% of the PPG signals measured by the wrist-type PPG BPM could be categorized as good (8% ± 3%) to excellent (6% ± 1%); the remaining signals were fair (86% ± 3%). The number of the signals obtained from the wrist-type PPG BPM categorized as fair and excellent were both significantly greater than those obtained from the upper-arm, cuffless PPG BPM (*p* = 0.029, * *p* < 0.05). Those categorized as good were not significantly different (*p* = 0.2).

### 3.3. Baseline and Long-Term Performance Assessment

**Figure 8:**
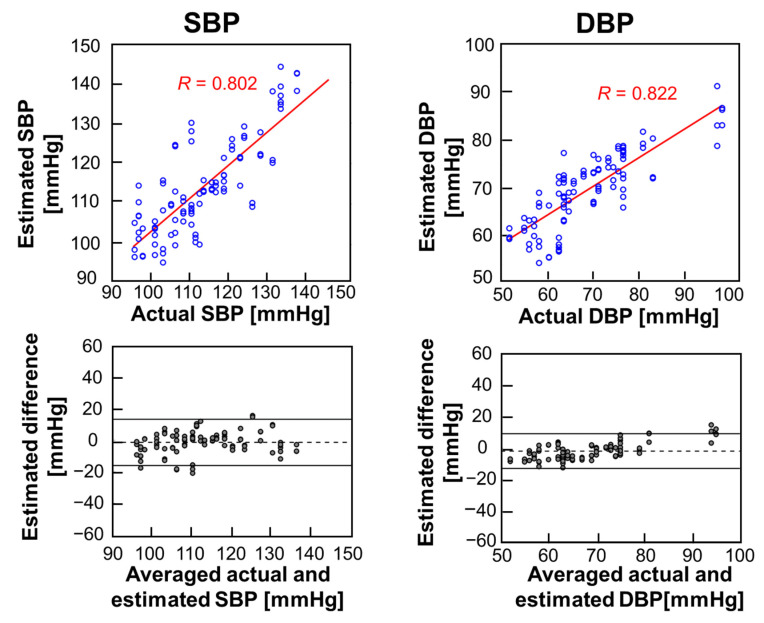
The correlation coefficient and Bland−Altman plot for ΔBP between actual BP and BP estimation with calibration. The SBP measurement with calibration showed excellent correlation (*r*-value = 0.802 and its associated *p* = 1.18 × 10^−22^), with a mean ΔBP of −1.373 mmHg (CI = −16.08 to 13.33) between the actual BP and the estimated BP. The DBP measurement with calibration also showed excellent correlation (*r*-value = 0.822 and its associated *p* = 9.91 × 10^−25^), with a mean ΔBP of −1.864 mmHg (CI = −12.88 to 9.15) between the actual BP measurement and the estimated BP.

The means ± SDs of the difference between the upper-arm, cuffless PPG BPM and reference cuffed BPM at week 1 (baseline) were −1.36 ± 7.24 and −2.11 ± 5.71 mmHg for SBP and DBP, respectively, which met the first criterion of ≤5 ± ≤8.0 mmHg (Table 2). The mean SD of the difference between the upper-arm, cuffless PPG BPM and the reference cuffed BPM at week 1 were 6.82 and 6.62 mmHg for SBP and DBP, respectively, which met the second criterion of SBP ≤ 6.89 mmHg and DBP ≤ 6.84 mmHg. Bland–Altman plots show the difference in SBP/DBP readings between the upper-arm, cuffless PPG BPM and the reference cuffed BPM (Figure 8); both SBP and DBP measured by the upper-arm, cuffless PPG BPM at week 1 showed excellent correlations with those measured by the reference BPM (*r* = 0.802 and 0.822, respectively; both *** *p* < 0.001) (Figure 8).

**Figure 9:**
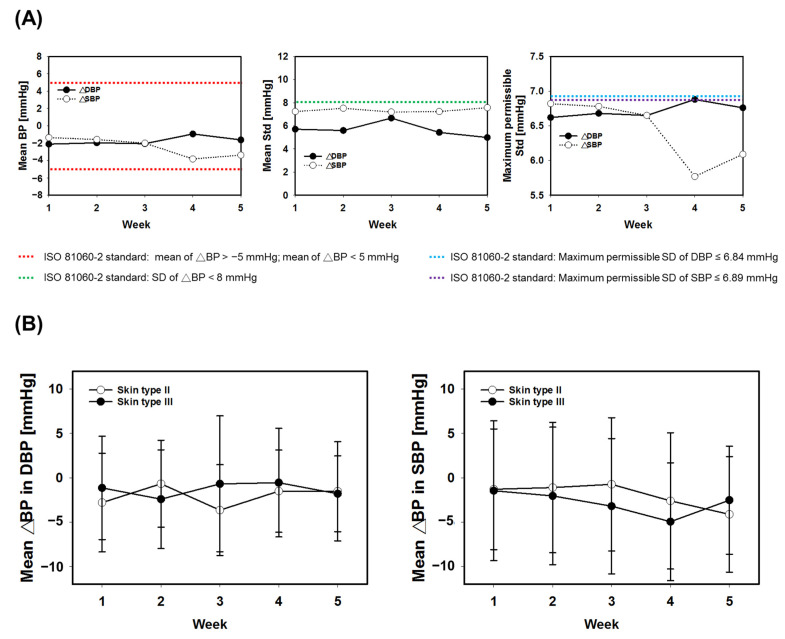
(**A**) Comparison of the ISO standard and cuffless BP estimation of long-term follow-up error, shown for the first week, second week, third week, and fourth week and for 1 month after the first-week calibration process, respectively. Dotted lines indicate ISO standard criteria (red dotted line: mean ∆BP; green dotted line: mean SD of ∆BP; blue dotted line: maximum permissible SD of DBP; purple dotted line: maximum permissible SD of SBP ≤ 6.89 mmHg). (**B**) Statistical comparison of mean ΔBP in SBP and DBP between the different skin phototypes (Fitzpatrick skin type II and type III). All results were not statistically different.

Table 2 lists the follow-up measurements from weeks 2–5 of the test and reference BP values in 30 subjects. The differences for uncalibrated SBP and DBP were −1.58 ± 7.52 mmHg and −2.06 ± 6.67 mmHg at week 2, respectively, and remained stable within the range of mean ± SD ≤5 ± ≤8.0 mmHg until week 5 (SBP/DBP = −3.38 ± 7.57 mmHg/−1.62 ± 4.99 mmHg) (Table 2 and Figure 9A). The mean SDs of the uncalibrated SBP and DBP were 6.82 mmHg and 6.62 mmHg at week 2, respectively, and remained stable within the range of SD ≤ 6.89 mmHg for SBP and ≤6.84 mmHg for DBP until week 5 (SBP and DBP = 6.09 and 6.76 mmHg) (Figure 9A). Similar results were obtained for populations with Fitzpatrick skin types II and III. The SBP and DBP between the test and reference devices were consistent within a range of mean ± SD ≤5 ± ≤8.0 mmHg from week 1 to week 5, and there was no difference in SBP or DBP between the two skin type groups (Figure 9B). Table 3 compares the uncalibrated BP changes over time to those measured by other studies that included follow-ups.

## 4. Discussion

In this study, the accuracy and precision of an upper-arm-designed, cuffless PPG BPM based on a nonlinear expGPR ML model architecture with a fine-tuning optimized method was evaluated by comparing its performance values to those of a reference cuffed BP device. The PPG signals obtained from the upper-arm, cuffless PPG BPM showed distinguishable systolic and diastolic peaks. The differences between the SBPs and the DBPs measured by the upper-arm, cuffless PPG BPM and the reference BPM at baseline and weekly follow-ups over 1 month satisfied the first criterion of ≤5 ± ≤8.0 mmHg and the second criterion of SBP ≤ 6.89 mmHg and DBP ≤ 6.84 mmHg described by the ISO 81060-2 standard [33].

Hypertension is a chronic condition that requires the proper management of BP. Many cuffless BPs have been recently developed for consumer use [15,34,35,41]. One commonly used technology to estimate BP in these cuffless BPMs is pulse transit time (PTT), which uses the difference in time between ECG and PPG signals to calculate BP [42]. However, PTT-based BPMs can interrupt the user’s sleep. Electrodes used to measure the ECG signal can also irritate the skin and decrease the quality of the ECG signal [43]. Another technology uses PPG signals to measure the pulse waveform at the wrist to estimate BP [30]. PPG-based BP measurement technology does not require the additional use of ECG, thereby eliminating the need for additional sensors and enabling continuous BP monitoring for 24 h or longer without disrupting everyday activity. However, most PPG-based cuffless BPMs are designed to be worn on the wrist [9,34,35,44], from which the signal obtained is less capable of representing the PPG pulse waveform than the signal obtained from the upper arm [25]. In this study, we used PPG technology to develop a cuffless BPM that measured brachial BP at the upper arm, resembling where traditional sphygmomanometers take readings (refer to Figure 2A). Our findings, as presented in Figure 6 and Figure 7, demonstrated that PPG signals acquired from the upper arm had better pulse waveform characteristics than those obtained at the wrist. The waveforms produced by our device were characterized by distinguishable systolic and diastolic peaks such as those obtained when measured by standard invasive BP monitoring procedures. In addition, our results aligned with previous literature indicating that green light upper arm modalities are less susceptible to micromotion artifacts (such as typing) compared with wrist-worn devices [45], thereby allowing for more accurate calculations of BP. Micromotion in daily life refers to small, unconscious movements of the body, such as fidgeting, shifting, or trembling. It can also refer to the small movements of joints and muscles during physical activity or normal function. Examples of micromotion in daily life include tapping a foot, shaking a leg, wriggling in a chair, or the twitching of the eyes. Despite the common use of red and infrared light in pulse oximeters [46] because of their deeper tissue penetration than green light [47,48,49], green light is often utilized in commercial wearable devices such as smartwatches because of vast amount of existing knowledge of the technology [50]. Previous research has shown that green light has a higher signal-to-noise ratio (SNR) and more favorable physiological measurement results in environments with motion [51,52].

Cuffless BPMs offer detailed insights into BP management to both patients and healthcare providers. Users can easily store their BP data and provide the data to their healthcare providers for informed treatment plans. Using cuffless BPMs to self-monitor BP may also promote healthy lifestyle changes such as daily BP measurements to control hypertension [2]. However, the accuracy and long-term performance of these devices are poorly characterized. The AAMI/ESH/ISO 81060-2 protocol established for cuffed BP devices does not apply to cuffless BPMs [10]. Additionally, many cuffless BPMs still require initial baseline calibration and regular recalibration with a cuffed BPM [9,10]. Specifically, the initial baseline calibration is used as a reference for estimating SBP and DBP; the estimation may vary over time if left uncalibrated. As shown by Yoon, the SD of the difference in BP values between the cuffless BPM and the reference BPM increased weekly [14], suggesting that the periodic calibration of cuffless BPMs is needed to prevent the decreased accuracy that occurs over time.

Given these limitations, ISO is creating a new standard specific to cuffless BPMs [6,53]. Researchers are also developing cuffless BPMs that do not require recalibration [14,54]. In the meantime, studies have adapted validation protocols to evaluate the performance of cuffless BPMs at different time points [9,10,13,14]. For example, Lee et al. [10] adapted a validation protocol from the ISO 81060-2 standard by adding repeated measurements after the first set of validations to evaluate BP changes over different time periods. This modification can inform how often recalibration should be performed after the initial calibration [10]. Vybornova et al. used this adapted validation protocol to assess the accuracy and precision of a wrist-worn, optical BPM (Aktiia bracelet) over a 1-month period; the overall accuracy of the initial and follow-up BPs measured by the Aktiia bracelet satisfied both criteria of the ISO 81060-2 standard [9]. The same adapted validation protocol with 1-month follow-up was also used to assess the accuracy of a smartphone-based BP monitoring app (AlwaysBP); however, the uncalibrated SBP and DBP measured at 3- and 4-week follow-ups slightly increased and failed to meet the accepted criterion of ≤5 ± ≤8.0 mmHg [14].

In this study, the differences between the uncalibrated BP values obtained from the upper-arm, cuffless PPG BPM and those obtained from the cuffed BPM from 4 weeks of follow-ups all satisfied both criteria of the ISO 81060-2 standard (Table 2 and Figure 9). These results indicate that the accuracy and precision of our device could be used to monitor continuous BP changes for long time periods. Understanding changes in uncalibrated BP values over different time periods guides manufacturers to establish proper calibration intervals for their devices (Table 3).

## 5. Conclusions

In this study, we designed a new device, an upper-arm, cuffless PPG BPM, called the WatchBP O3 wearable, to measure BP. The proposed prediction model was based on a nonlinear expGPR ML model with a fine-tuning optimization method, which enhanced model training and reduced the size of the model. A fully automated calibration system from the same manufacturer was also integrated to increase the precision of the calibration model and user convenience. The upper-arm, cuffless PPG BPM used in this study measured BP from the upper arm, a body part that was less affected by motion artifacts than the wrist, and more comfortable compared with wearing a device on the fingers. Additionally, compared with the PPG signals obtained from the wrist, the PPG waveforms obtained from the upper arm showed higher-quality, distinguishable systolic and diastolic peaks such as those measured by standard invasive BPMs. The accuracy and the precision of an upper-arm, cuffless PPG BPM were evaluated by comparing its performance with that of a reference cuffed BPM according to the ISO 81060-2 standard. The initial calibrated BP and the follow-up uncalibrated BP measured weekly over a 1-month period by the upper-arm, cuffless PPG BPM met the requirement criteria specified by the ISO 81060-2 standard, which required a BP ≤ 5 ± ≤ 8.0 mmHg as well as an SBP ≤ 6.89 mmHg and a DBP ≤ 6.84 mmHg. Most PPG BPMs require an initial calibration using a traditional cuffed BPM that inconveniently must be regularly recalibrated to ensure accuracy. Therefore, we assessed changes in uncalibrated BP values of our PPG BPM over different time periods, which could help manufacturers determine proper calibration intervals. Overall, the cuffless PPG BPM with the upper-arm design evaluated here provided high-quality PPG signals and an efficient prediction model within an embedded system; its satisfactory accuracy and its precision over a 1-month period render it a convenient and practical alternative for long-term BP monitoring.

There were limitations to this study. First, the number of subjects recruited in this study did not meet the requirements of the standard (ISO 81060-2), so it should not be considered a clinical validation study for the cuffless BPM. Another limitation was that the skin tone of the subjects was in the lighter skin range (Fitzpatrick types II and III), and thus, caution should be exercised when generalizing the current results to a population with darker skin tones. Lastly, the population in the current study was generally healthy. Further studies should be conducted on a variety of patient populations to test the clinical feasibility of the upper-arm cuffless PPG BPM.

## Figures and Tables

**Table 1:** Subject characteristics and their corresponding weekly statistics of measured BP in 5 weeks.

	n [%]	Mean ± SD	Range
Age [year]	30 [100%]	26.3 ± 5.3	20.0–42.0
Female gender	11 [36.67%]		
Male gender	19 [63.33%]		
Arm circumference [cm]	30	27.2 ± 2.8	22.0–31.0
Cuff size M–L [22–31 cm]	30 [100%]		
Skin phototype [Fitzpatrick skin type]	II, Ivory = 16 [53.33%] III, Beige = 14 [46.67%]		
**Long-term BP distribution [n = 90]**	**SBP, mmHg [Mean ± SD]**	**DBP, mmHg [Mean ± SD]**	
Week 1	112.2 ± 11.6	67.7 ± 9.8	
Week 2	110.7 ± 12.1	65.7 ± 9.5	
Week 3	110.3 ± 13.8	66.8 ± 11.1	
Week 4	110.4 ± 12.5	67.6 ± 10.0	
Week 5	109.8 ± 12.0	66.7 ± 8.7	
Total	110.7 ± 12.4	66.9 ± 9.8	

**Table 2:** Performance evaluation for ∆BP and the cumulative percentage of ∆BP over a 1-month period (5 weeks).

Cumulative Percentage of ∆BP [%]					
	Grading Criteria	≤5 [%]	≤10 [%]	≤15 [%]	∆BP [mmHg]
Week 1 (baseline)	DBP	57.29†	92.71#	98.96#	−1.86 ± 5.50
	SBP	60.42†	82.29†	92.70†	−1.37 ± 7.35
Week 2	DBP	65.59#	91.40#	95.70#	−1.96 ± 5.60
	SBP	56.99†	81.72†	91.40†	−1.58 ± 7.52
Week 3	DBP	53.76†	88.17†	96.77#	−2.06 ± 6.67
	SBP	50.54†	79.57†	91.40#	−2.00 ± 7.20
Week 4	DBP	64.52#	93.55†	98.92#	−0.94 ± 5.43
	SBP	53.76†	80.65†	90.32†	−3.82 ± 7.24
Week 5 (1 month)	DBP	66.67#	93.55#	100.0†	−1.62 ± 4.99
	SBP	36.56*	74.19*	93.55†	−3.38 ± 7.57

The symbols *, †, and # indicated the BHS grading of A, B, and C, respectively. BHS grading criteria (mmHg, cumulative percentage): Grade A (≤5, 60%; ≤10, 85%; and ≤15, 95%), Grade B (≤5, 50%; ≤10, 75%; and ≤15, 90%), Grade C (≤5, 40%; ≤10, 65%; and ≤15, 85%), and Grade D (worse than Grade C). ISO 81060-2 standard: ΔBP < 5 mmHg, mean SD < 8 mmHg.

**Table 3:** Comparison of estimation errors and maximum calibration interval of the upper-arm-designed, cuffless PPG with those of other studies.

Reference	Devices	Subjects [n]	Max. Calibration Interval	Estimation Errors [Mean ± SD]	
				SBP	DBP
Current Study	Microlife WatchBP O3 wearable	30	1 month	−3.38 ± 7.57	−1.62 ± 4.99
Yoon et al. (2022) [14]	AlwaysBP	15	1 month	0.1 ± 8.8	−2.4 ± 7.6
Vybornova et al. (2021) [9]	Aktiia bracelet	86	1 month	0.46 ± 7.75	0.39 ± 6.86
Miao et al. (2017) [40]	MLR- and SVR-based BP models	10	6 months	−1.267 ± 5.98 (MLR) −1.148 ± 5.79 (SVR)	−1.38 ± 5.49 (MLR) −1.194 ± 5.29 (SVR)

Estimation error: absolute BP difference between the test and reference devices. MLR: multivariate linear regression. SVR: support vector regression.

## Data Availability

The datasets generated for this study are available on request to the corresponding author.
